# The impact of CPR coach presence and position on team leader and team performance during asystole simulation scenario: a randomized simulation-based trial

**DOI:** 10.1371/journal.pone.0344568

**Published:** 2026-03-12

**Authors:** Giulia Mormando, Emanuele Capogna, Ilaria Costantini, Sandro Savino, Anna Vittadello, Marina Campione, Annalisa Boscolo, Silvia Bressan, Paolo Navalesi

**Affiliations:** 1 Department of Medicine (DIMED), University of Padova, Padova, Italy; 2 EESOA Simulation Center, Rome, Italy; 3 UOC Pronto Soccorso Ospedale Cazzavillan Arzignano ULSS 8 Berica Vicenza, Vicenza, Italy; 4 UOC Pronto Soccorso Azienda Ospedale Università di Padova, Padova, Italy; 5 Faculty of Economics and Law, University of Enna Kore, Enna, Italy; 6 Institute of Anaesthesia and Intensive Care, Padova University Hospital, Padova, Italy; 7 Department of Women’s and Children’s Health (SDB) – University of Padua, Padova, Italy; University of Palermo, ITALY

## Abstract

**Background:**

High-quality cardiopulmonary resuscitation is critical for patient survival, and its effectiveness depends on compression quality, team coordination, and leadership. The CPR coach provides real-time feedback aimed at optimizing chest compressions and enhancing the overall resuscitation process. The primary aim is to evaluate whether the presence and the position of a CPR coach are equivalent compared to the absence of a CPR coach regarding the team leader’s performance. As a secondary objective, it was investigated whether the presence and the position of the CPR coach influence the teams’ performance.

**Methods:**

This is single-center, randomized, controlled, three-arm, simulation-based equivalence trial. Teams of residents participated in standardized asystole scenarios, randomized to one of three groups: Group A (CPR coach positioned near the defibrillator), Group B (CPR coach allowed to move freely), and Group C (no CPR coach). The primary outcome was assessed using the Resuscitation Team Leader Evaluation Scale. The secondary outcome was measured by the Clinical Performance Tool and a CPR execution quality score derived from simulation manikin feedback.

**Results:**

A total of 42 teams were included in the analysis. With respect to the primary outcome, team leader performance was statistically equivalent across Groups A, B, and C. As for the secondary outcome, no significant differences were observed in overall team performance.

**Conclusion:**

The presence and position of a CPR coach did not significantly influence team leader or team performance. Further research is needed to explore the role of CPR coaches in real-life settings and among more experienced teams.

**Trial registration:**

Trial registration: NCT05309434. Registered 15/04/2022.

## Background

Cardiopulmonary resuscitation (CPR) is a complex task requiring rapid decision-making and coordination. The team leader must manage multiple responsibilities, including overseeing team members, identifying reversible causes, and ensuring high-quality chest compressions (CC), a key factor in survival [1]. Compliance with American Heart Association (AHA) guidelines improves with CPR feedback, and real-time visual feedback has been shown to enhance compression consistency [2].

Recently, the role of the CPR coach has been introduced as a provider offering real-time feedback to optimize chest compression quality, ensure guideline adherence, minimize pauses, and support the team leader [3]. Studies suggest this role improves adherence to advanced life support protocols [4] and providers’ perception of compression depth [5]. While it may reduce workload for CPR providers, its impact on team leader performance remains unclear [6,7]. Whether the presence and spatial positioning of the CPR coach interfere with the performance of the Team Leader and the team’s work remains to be substantiated [8–11].

### Objectives

Simulation was used as an investigational research method. The primary aim of this study was to evaluate whether the presence and position of a CPR coach are equivalent to the absence of a CPR coach in terms of team leader performance during simulated CPR scenarios. Two CPR coach positions were tested: stationary near the defibrillator or moving freely within the simulation room. As a secondary objective, the study investigated whether the presence and position of the CPR coach influence team performance.

## Methods

### Study design and setting

This is a single-centre (monocentric), randomized controlled, three-arm parallel-group simulation-based trial with an equivalence design, conducted in an off-site intermediate-fidelity simulation setting. It was registered at Clinical. Trial. Gov (ID n. NCT05309434) and was approved by the Ethics Committee of Azienda Ospedale Università di Padova (approval n 0001472). The study was reported according to the extended Consolidated Standards of Reporting Trials (CONSORT Extended) reporting guidelines for health care simulation-based research. (S1 File) [12].

The study was carried out between May and December 2023 at “SIMULARTI,” the Medical Simulation Center of the Department of Medicine (DIMED) of the University of Padua, Padua, Italy. The analysis was performed between March and November 2024.

### Participants

Residents in emergency medicine, internal medicine, anaesthesiology and critical care, and from the first to third postgraduate year (PGY1-2-3) of residency programs at the University of Padua (Padua, Italy) were recruited by a researcher by email after providing written informed consent. Only participants who had completed the AHA’s international certifications -the Basic Life Support (BLS) or Advanced Cardiac Life Support (ACLS) during residency prior to participation in the simulation sessions – were considered eligible and recruited in May 2023. Exclusion criteria was personal leave during the study period. All participants provided written informed consent, and their privacy, confidentiality, and anonymity were fully guaranteed.

### Randomization, allocation concealment, and blinding

Randomization was performed by an independent study statistician and completed prior to the simulation sessions. After enrolment, each participant was assigned a unique anonymized study identification number. First, participants were randomly allocated to teams, each consisting of four study participants and two instructors acting as standardized nurses. Second, roles within each team were randomly assigned. In the intervention groups (A and B), participants were allocated to the roles of team leader, two team members, and CPR coach; in the control group (C), participants were assigned to the roles of team leader and three team members. Third, once team composition and role assignment were completed, teams were randomized in a 1:1:1 ratio to one of the three study arms: Group A (CPR coach positioned near the defibrillator), Group B (CPR coach allowed to move freely within the simulation room), or Group C (no CPR coach). Allocation concealment was ensured using opaque sealed envelopes, externally labelled with the team identification number and containing the assigned study arm. Envelopes were opened immediately before the start of each simulation session. Due to the nature of the intervention, blinding of participants, simulation instructors, and video reviewers was not feasible. However, data analysis was conducted by the study statistician, who was blinded to group allocation.

### Interventions

Each team conducted a 10-minute asystole standardized simulation scenario in a dedicated simulation room at the Simulation Center of the University of Padova (S2 File). The scenario represented an adult patient presenting to the emergency department in cardiac arrest with asystole as the initial and persistent rhythm, regardless of the resuscitation manoeuvres performed.

For the scenario a Trauma Hal mannequin (Gaumard Scientific) and a Zoll R series defibrillator characterized by visual feedback on the rate, depth, release of compressions and the monitoring to end tidal CO2 were used.

Only trainees assigned to the CPR coach role in the intervention groups received additional standardized CPR coach training. This training consisted of a one-hour session based on the methodology described by Cheng et al., focusing on real-time feedback, communication strategies, and optimization of chest compression quality [13].

In Group A, the CPR coach was positioned near the defibrillator throughout the simulation. In Group B, the CPR coach was free to move around the simulation room. In the control group (C), the simulation was conducted without a CPR coach.

### Outcomes

#### Primary outcome.

For the primary outcome, Resuscitation Team Leader Evaluation Scale was used to assess leadership and communication skills, medical knowledge and clinical skills and leader’s overall performance **(S3 File)** [14].

#### Secondary outcome.

For the secondary outcome, the team performance was evaluated by the CPT (Clinical Performance Tool) **(S4 File)** [15] and by CPR execution quality score.

#### Outcome assessment.

Concerning the CPT, a global score including the sum of the individual items relating to quality, timing, and the sequence of specific actions (except the “pulse recheck after return of spontaneous circulation, ROSC”) was used to evaluate the team performance. Each item in the CPT was rated from 0 (not performed) to 2 (adequately performed) except for the score of defibrillation (score from 0 to 1) based on prior studies that validated this scoring system for evaluating clinical performance in high-stress simulation environments [15]. The maximum possible score of 11 reflects the sum of essential resuscitation tasks critical for team performance during CPR scenarios.

The CPR execution quality score was assessed by the software of Trauma Hal mannequin (Gaumard Scientific) based on the following criteria: chest compression fraction >60% (minimizing interruptions), compression rate between 100 and 120/min, compression depth between 50 and 60 mm, and full chest recoil >75%. Each criteria, if it was met, was given a score of 1. The sum of the criteria resulted in a score ranging from 0 to 4.

### Study procedures

All simulations were video recorded, and the leader’s performance was evaluated by three trained expert video reviewers. Inter-rater reliability was assessed by repeated testing until satisfactory inter-observer reliability (Cohen’s Kappa value >0.70) [16,17] was achieved.

### Data collection and management

Participants’ data were anonymized using unique identifiers, and all identifying information was removed before data analysis. Confidentiality was strictly maintained in accordance with institutional guidelines, and no personal data were shared beyond the research team. All data are checked for completeness and accuracy by the principal investigators. All data is securely stored in electronic databases, accessible only by permission of principal investigator by institutional account. Only the principal investigator and the study statistician had access to the final trial dataset. Data was analysed by the study statistician who was blinded to group.

### Statistical analysis

#### Sample size calculation.

The sample size was determined based on the primary endpoint within an equivalence framework. An equivalence margin of η² = 0.10 was defined a priori as the maximum between-group effect size considered compatible with practical equivalence in Team Leader performance.

Statistical power was estimated using Monte Carlo simulation under the planned study conditions. The simulations indicated that a minimum of 33 teams (11 per group) would provide approximately 80% power to demonstrate equivalence at a two-sided α level of 0.05. To account for potential data loss or non-evaluable teams, the planned sample size was increased by approximately 30%.

#### Data analysis plan.

Statistical calculations were performed using R software version 4.3.0. Quantitative data were summarized using appropriate descriptive statistics (i.e., mean, standard deviation, median, minimum, and maximum). Results were expressed as mean ± SD or median with interquartile range. Qualitative data were summarized by absolute and relative frequency distribution and expressed as number of events [%]. The normality of the data was evaluated using the Kolmogorov-Smirnov and Shapiro-Wilk tests. In cases where normality assumptions were violated, non-parametric tests were used. Descriptive statistics were utilized to summarize the scores from the complete ‘Resuscitation Team Leader Evaluation’ instrument, as well as from its two subscales: ‘leadership and communication skills’ (LCS) and ‘knowledge and clinical skills’ (KCS) [18]. To assess the equivalence of resuscitation Team Leader performance between different groups, a equivalence test based on η2 parameter of the ANOVA model was applied. The Kruskal Wallis test was used to evaluate whether the CPT score achieved in performing the control procedures varied between individual groups. Confidence intervals were reported where applicable, and p-values below 0.05 were considered statistically significant.

## Results

**Fig 1:**
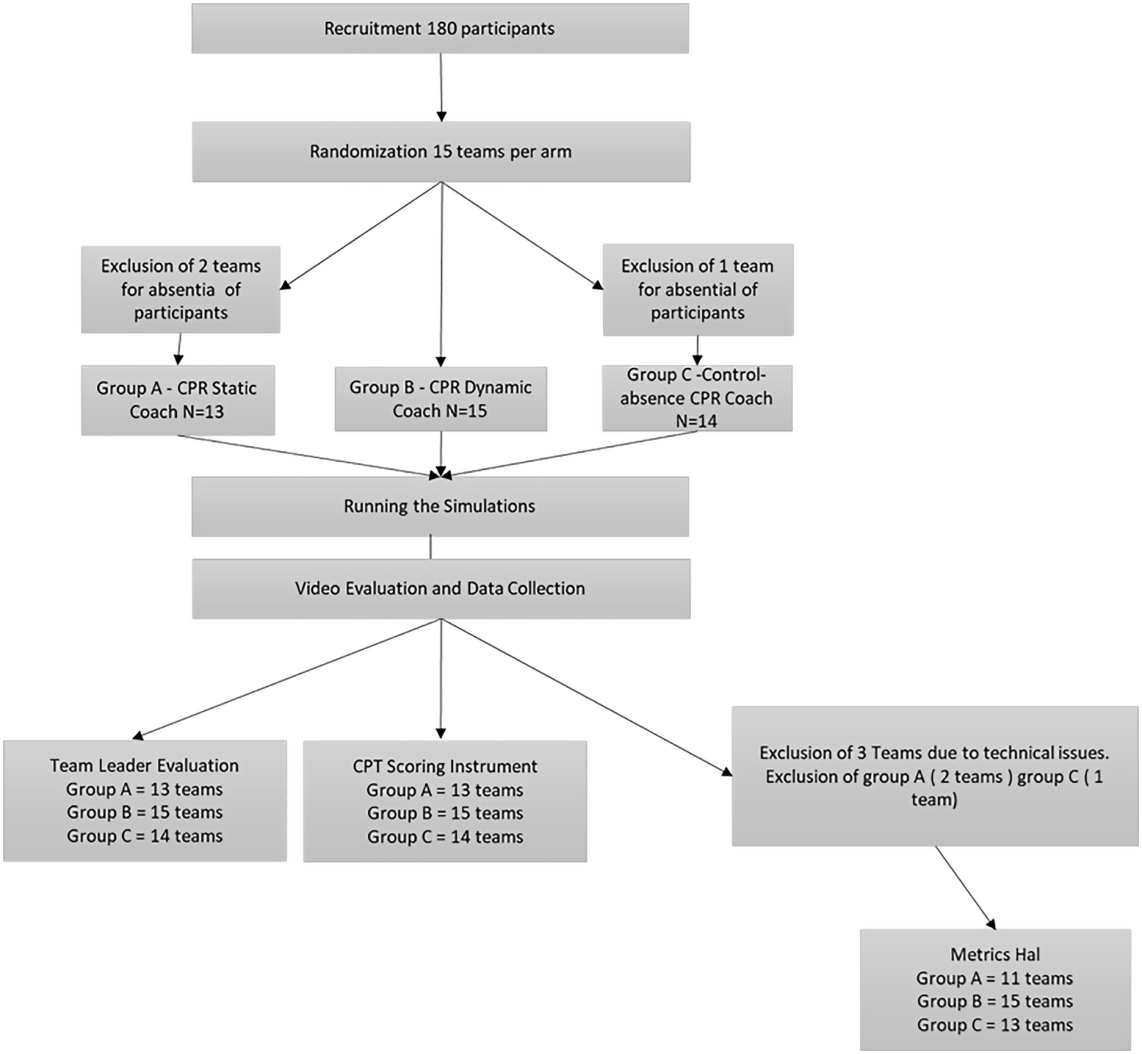
Trials diagram.

Between May and December 2023, a total of 180 participants were enrolled and randomly assigned to Groups A, B, and C. Owing to the absence of some participants during the simulation sessions, a total of 42 teams were ultimately formed, comprising 168 participants. Specifically, Group A included 13 teams, Group B 15 teams, and Group C 14 teams. Due to technical problems of the manikins the variable CPR execution quality score could not be collected in 3 teams. Regarding all other variables, data were collected from all teams in the study. **Fig 1** shows Trials Diagram. Demographic data are shown in **Table 1**. All had previous simulation experience through AHA courses.

**Table 1:** Characteristics of individual participants.

	N	%
**Age, median** **(range, 25–75%)**	29.0 (28.5 - 30.5)	
**Year of specialisation, median (range, 25–75%)**	2 (2.0-2.0)	
**Type of school**		
Emergency medicine	87	52
Anesthesia and critical care	64	38
Internal medicine	17	10
**Certification**		
ACLS	11	6.6
BLS	130	77.3
BLS and ACLS	27	16.1

Values are reported as median (interquartile range, 25th–75th percentile) or number (%), as appropriate. BLS = Basic Life Support; ACLS = Advanced Cardiac Life Support.

### Primary outcome

Regarding the primary outcome, that was evaluated through the questionnaire “Resuscitation team leader evaluation scale” **(S3 File)** [14] the performance of the team leader was statistically equivalent (p < .05) in the A, B, C groups examined. Consequently, the presence and position of the CPR coach did not affect the Team Leader Performance.

**Fig 2:**
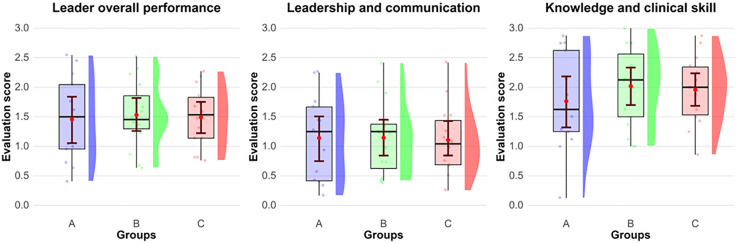
Resuscitation team leader evaluation tool scores Resuscitation Team Leader evaluation for the three different groups. A: CPR coach near defibrillation, B: CPR moving around, C: without CPR coach. The competencies of Team Leader analyzed were assessed in terms of leadership and communication.

**Fig 2** describes the correlation between groups A, B, C and the resuscitation team leader evaluation through the two different subscales evaluated “leadership and communication” and “knowledge and clinical skills” and the evaluation in its entirety called “Overall Performance”.

The result of “Overall Performance,” “leadership and communication,” and “knowledge and clinical skills” were respectively for group A of 1.5 (sd 0.7), 1.1 (sd 0.7) and 1.8 (sd 0.8); for group B of 1.5 (sd 0.5), 1.1(sd 0.6) and 2 (sd 0.7); for group C 1.5(sd 0.5), 1.1 (sd 0.6) and 2 (sd 0.6). These results are shown in **Table 2** where the equivalence between the three arms examined in the study is highlighted.

**Table 2:** Team leader performance scores and equivalence testing across study groups.

Team Leader	Group	Mean (±SD)	Equivalence Bound (η^2^)	p.value
Overall Performance	A (13)	1.5 (±0.7)	<0.01	P = 0.0431
	B (15)	1.5 (±0.5)		
	C (14)	1.5 (±0.5)		
Leadership and communication	A (13)	1.1 (±0.7)	<0.01	P = 0.0117
	B (15)	1.1 (±0.6)		
	C (14)	1.1 (±0.6)		
Knowledge and clinical skill	A (13)	1.8 (±0.8)	<0.01	P = 0.0481
	B (15)	2 (±0.7)		
	C (14)	2 (±0.6)		

Values are reported as mean (standard deviation). Group A: CPR coach positioned near the defibrillator; Group B: CPR coach moving freely within the simulation room; Group C: no CPR coach. Equivalence testing was performed using a predefined equivalence bound (η² < 0.01). p-values refer to equivalence tests.

### Secondary outcome

**Fig 3:**
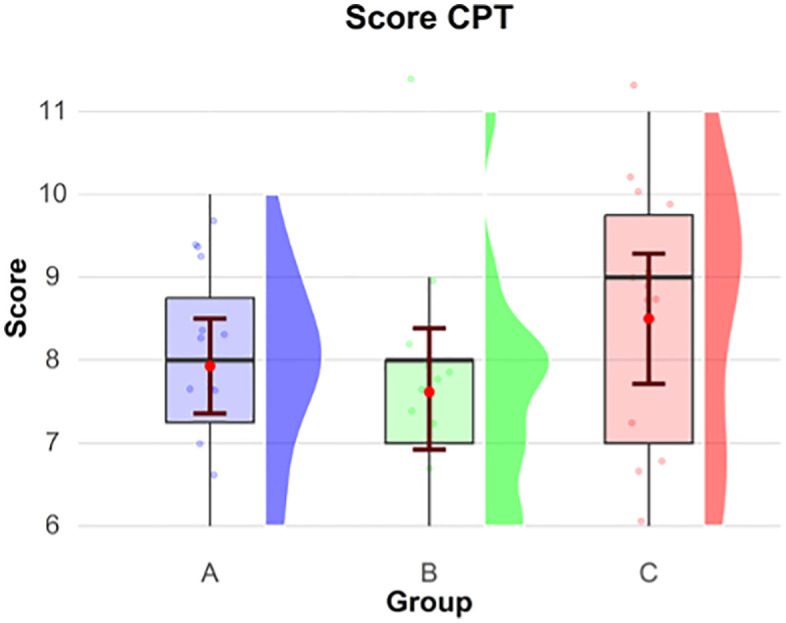
Score CPT by groups A: CPR coach near defibrillation, B: CPR moving around, C: without CPR coach.

The secondary outcome aimed to assess team performance among the three groups evaluated in the study, using the CPT and CPR execution quality score. The CPT assessment is described in **Fig 3** where there was no a statistically significant difference between the three groups (p > .05). The mean of the scores was for group A of 8 (±1), for group B 8 (±1), and for group C 9 (±1) visible in the **Table 3**.

**Table 3:** Clinical performance tool (CPT) scores across study groups.

Group	N	Mean (±SD)	p-value
A	13	8 (±1)	
B	15	8 (±1)	P = 0.411
C	14	9 (±2)	

Values are reported as mean (standard deviation). Group A: CPR coach positioned near the defibrillator; Group B: CPR coach moving freely within the simulation room; Group C: no CPR coach. Group comparisons were performed using the Kruskal–Wallis test.

As for the data obtained from the manikin feedback, 3 teams were excluded due to mechanical issues. The results of CPR execution quality score show that again there was no statistically significant difference (p > 05). The median values were 3 [IQR 2–3] for Group A (n = 11), 3 [IQR 2–4] for Group B (n = 15), and 2 [IQR 2–3] for Group C (n = 13). It can be seen from these data that team performance is not affected by the presence and position of the CPR coach either.

## Discussion

The introduction of a CPR coach may represent a significant innovation in cardiopulmonary resuscitation management, with the potential to enhance the quality of chest compressions and improve team coordination [3]. The AHA emphasizes the importance of combining clinical competence with leadership and communication skills [19,20], and several studies have confirmed the positive impact of these non-technical skills on resuscitation performance [21–26].

In this simulation-based study, Team Leader performance was statistically equivalent across the groups studied and overall team performance did not have a significant impact, regardless of whether the CPR coach was mobile or positioned near the defibrillator. Similar findings were reported in a pilot study that examined the impact of a novice CPR coach during paediatric cardiac arrest simulations, where no improvement in team performance was observed. However, the authors themselves acknowledged that the limited sample size and lack of adequate coach training likely influenced these outcomes negatively [27]. In this study, although the sample size was adequate, all participants were junior residents in the early years of their training. It is plausible that, despite some clinical experience, they had not yet received structured and sufficient leadership training. This could explain the absence of significant improvements in team performance, even in the presence of both a team leader and a CPR coach. This hypothesis aligns with findings from Høyer et al. [28], who reported that junior physicians often struggled with effective delegation and communication during CPR and called for more targeted leadership training, including guidance on verbal behaviours.

It is also possible that the CPR coach can express their full potential only in experienced and well-structured teams, where interprofessional communication is already established and the coach can truly act as a facilitator rather than an additional element to be integrated. Another factor to consider is that the participants were already under high cognitive load due to the demands of high-fidelity simulation. In such settings, the introduction of an additional figure not fully integrated into the communication flow may have created confusion rather than clarity, potentially undermining the expected benefits.

However, a recent systematic review [29] identified seven studies, mostly conducted in simulated paediatric settings, suggesting that incorporating a CPR coach into resuscitation teams may improve CPR quality, reduce interruptions, and enhance adherence to guidelines compared to teams without a coach. In particular, coached teams demonstrated a higher fraction of effective chest compressions and shorter pauses, especially when coaches were formally trained and when real-time feedback devices were used. Despite these encouraging findings, the review also concluded that the overall certainty of the evidence remains low to very low, due to methodological biases, clinical heterogeneity, and the lack of randomized controlled trials in real-life clinical settings. Notably, no evidence was found regarding patient-centered outcomes such as return of spontaneous circulation (ROSC), survival to hospital discharge, or favorable neurological recovery. Taken together, our findings and the broader literature suggest that the CPR coach may be more effective in experienced and well-trained teams operating in technologically supported environments. In training contexts or resource-limited settings, however, the potential benefits of the CPR coach may be limited or even negligible. Furthermore, the coach’s role appears to have a greater impact on task coordination and minimizing CPR pauses than on reducing the cognitive workload of the team leader, a conclusion also supported by the review [29].

In this study, the effect of the CPR coach’s position on team and team leader performance were examined. The results showed no significant differences between groups with mobile coaches versus those positioned next to the defibrillator. The analysis was based on direct observation of recorded scenarios; however, more sophisticated tools may allow for a deeper understanding of team dynamics. A promising example is provided by Petrosoniak et al. [11], who developed a workflow-tracking tool capable of generating visual representations of participants’ movements and interactions during simulation.

Finally, it is important to note that the effectiveness of a CPR coach may depend not only on their physical position or individual training, but also on the ability of the team leader to integrate them into the decision-making process. In this sense, joint training programs for team leaders and CPR coaches, focused on communication and strategic coordination, could represent a valuable direction for future research and educational development [30].

Ultimately, the effectiveness of CPR coaching likely depends on how the role is implemented, whether as a dedicated new team member, a dynamic rotating role, or an integrated task for existing staff. Future studies should explore integrated training models, more precise cognitive load measures, and real-world clinical trials to determine how best to harness the potential of the CPR coach in everyday practice.

During the revision of this manuscript, updated American Heart Association (AHA) guidelines for cardiopulmonary resuscitation were published in October 2025. While these guidelines introduce refinements in resuscitation education and implementation strategies, the core principles of high-quality CPR, team leadership, and effective communication remain consistent with previous recommendations. As such, the main findings of this study—focused on team leader performance, team dynamics, and the role of the CPR coach—are unlikely to be substantially affected by the updated guidelines. Nevertheless, future research should evaluate CPR coaching models within the framework of the most recent AHA recommendations, particularly as new educational and technological approaches are increasingly emphasized.

### Limitations

This study has several limitations. First, all members of the resuscitation team were residents from different postgraduate years (PGY1–2–3). Differences in clinical experience and exposure to resuscitation scenarios may have influenced leadership behaviours, cognitive load, and team coordination, potentially attenuating the measurable effect of the CPR coach. More junior residents may have focused primarily on task execution, while more senior residents may have had greater situational awareness and leadership skills, introducing heterogeneity in team performance. Second, in real-life scenarios, resuscitation teams are typically multiprofessional and may be required to operate in more challenging environments—characterized by background noise, the presence of multiple patients, or bystanders. These factors could lead to more complex interactions between the CPR coach, the team leader, and other team members. Third, this was a single-centre simulation-based study, which may further limit the generalisability of the results to other institutions, training programmes, or healthcare systems with different organisational structures and educational practices.

While it is believed that this study can help stimulate further research into the role and optimal positioning of the CPR coach in simulated resuscitation scenarios, it is also recognized that real-life studies are likely needed to validate the effectiveness of the CPR coach model.

## Conclusions

In the study, the introduction of CPR coaches—regardless of whether they were positioned next to the defibrillator or allowed to move freely within the resuscitation room—did not significantly impact the performance of the team leader or the overall team.

Further simulation-based and clinical studies are warranted to better understand the dynamics of interaction between the CPR coach and the team leader, and to explore how this relationship might be optimized to improve patient outcomes.

## Supporting information

S1 File Consort checklist. (DOCX)

S2 File Scenario. (DOCX)

S3 File Resuscitation team leader evaluation. (PDF)

S4 File CPT clinical performance tool. (PDF)

S5 File Ethics committee. (PDF)

S6 File Protocol. (PDF)

S7 File Traduction of protocol. (PDF)

## Abbreviations

ACLS: advanced cardiac life support
AHA: American Heart Association
BLS: Basic life support
CPR: Cardiopulmonary resuscitation
CC: Chest compressions
CPT: Clinical Performance Tool
ETT: Endotracheal tube
ECG: Electrocardiography
EM: Emergency Medicine
JIT: Just-in-Time
IO: Intraosseous
IV: Intravenous
med: Medicine
PALS: Pediatric advanced life support
PGY1-3: Postgraduate 1-2-3 year
ROSC: Return of spontaneous circulation
