# Supplementary material for: The impact of CPR coach presence and position on team leader and team performance during asystole simulation scenario: a randomized simulation-based trial
Source: PLoS One. 2026 Mar 12;21(3):e0344568. doi: 10.1371/journal.pone.0344568 (PMC12981441; doi:10.1371/journal.pone.0344568)
Supplement: S2 File — (DOCX) [file pone.0344568.s002.docx]

Clinical case

pre-case indications: patient presenting to emergency department for malaise with sense of fainting, pale cold sweat.

The team is taken to the simulation room (shock room) when the patient arrives, the team's entry into the shock room coincided with the start of the simulation. Total simulation time 10 minutes.

Initial patient presentation: cardiac arrest with asystole presentation rhythm, regardless of the maneuvers performed the patient remains throughout the simulation in cardiac arrest with asystole rhythm

Material present in shock room:

- Stretcher with patient: Trauma Hal mannequin (Gaumard Scientific)
- - Multiparameter monitor ( with electrode capability, pressure measurement, saturation measurement)
- Zoll R series defibrillator characterized by visual feedback on the rate, depth, release of compressions and the monitoring to end tidal CO2 It presented both manual and self-adhesive plates
- - Emergency cart with medications (Adrenaline, amiodarone, lidocaine, propofol, midazolam, succinylcholine, Rocuronium NaCl 0.9%, etc.), simple and advanced ventilation aids, etc..
