# Supplementary material for: The impact of CPR coach presence and position on team leader and team performance during asystole simulation scenario: a randomized simulation-based trial
Source: PLoS One. 2026 Mar 12;21(3):e0344568. doi: 10.1371/journal.pone.0344568 (PMC12981441; doi:10.1371/journal.pone.0344568)
Supplement: S3 File — (PDF) [file pone.0344568.s003.pdf]

FACULTY OF MEDICINE | UNIVERSITY OF CALGARY

Resuscitation Team Leader Evaluation

STUDY NUMBER	ASSESSOR

Example ○ ● Please use an ink pen

Please rate the team leader on the performance statements according to the following scale.
Please use 'N/A' if the statement is not relevant to the scenario.

Part I: Leadership and Communication Skills

The team leader...	Not Performed/ Not Observed 0	Performed, but ineffectively, incompletely or inconsistently 1	Performed adequately most of the time 2	Performed well consistently 3	Item not relevant to scenario N/A
1. **Clearly identifies he/she will lead the resuscitation**	○	○	○	○	○
2. **Delegates roles and responsibilities to team members**	○	○	○	○	○
3. **Maintains control of leading the resuscitation**<br>- manages distractions<br>- avoids allowing others to give orders<br>- controls noise and crowd	○	○	○	○	○
4. **Uses effective closed loop communication**<br>- questions and orders are given clearly and assertively<br>- person spoken to is identified by name or other clear method<br>- ensures team member heard and understood (e.g. through member's response to question or by asking for verbal confirmation once order completed)	○	○	○	○	○
5. **Manages team resources and distributes workload appropriately**<br>- avoids overloading or underloading team members<br>- avoids giving multiple orders at once<br>- prioritizes multiple orders when several are needed	○	○	○	○	○
6. **Verbalizes thoughts and summarizes progress periodically for benefit of team (shares situational awareness and mental models)**<br>- describes events so far<br>- states suspected diagnosis as well as other possibilities	○	○	○	○	○
7. **Asks for and acknowledges input from team members**<br>- asks for other ideas<br>- asks for confirmation of suspected diagnosis<br>- incorporates ideas from team when appropriate	○	○	○	○	○
8. **Reassesses and reevaluates situation frequently**<br>- verbally identifies changes in patient status in a timely fashion<br>- acknowledges changes in status identified by team members	○	○	○	○	○
9. **Avoids fixation errors (getting "stuck" on a particular issue)**<br>- acknowledges information that is inconsistent with interpretation<br>- uses new information or changes in status as an opportunity to reconsider other diagnoses<br>- reassesses situation when interventions not producing desired effect	○	○	○	○	○
10. **Refrains if possible from active participation (hands-off)**	○	○	○	○	○
11. **Shows anticipation of future events by asking for preparation of equipment or medication not yet needed**<br>- asks for x-ray to be called ahead of when ready<br>- asks for infusions to be mixed up before needed	○	○	○	○	○
12. **Asks for appropriate help early and shows awareness of own limitations.**<br>- asks for additional personnel for extra hands<br>- asks for consultants to be called for advice	○	○	○	○	○

34660

Page 1 of 2

Fig. 1. Pediatric resuscitation team leader evaluation tool tested in Phase II.

Part II: Knowledge and Clinical Skills

The team leader...	Not Performed/ Not Observed 0	Performed, but ineffectively or incompletely 1	Performed effectively but delayed or out of sequence 2	Performed well in a timely manner 3	Item not relevant to scenario N/A
1. **Obtains preliminary history quickly or designates other to do so**	O	O	O	O	O
2. **Obtains full cardiorespiratory monitoring and full set of vitals promptly**<br>- HR, RR, BP, Sat, Temp	O	O	O	O	O
3. **Obtains assessment of airway patency and protection**<br>e.g. - establishes if patient verbalizing<br>- checks for open airway<br>- identifies protection of airway based on neurological status	O	O	O	O	O
4. **Obtains assessment of breathing adequacy**<br>- auscultation<br>- assessment of work of breathing<br>- adequacy of ventilation (rate, depth)	O	O	O	O	O
5. **Asks for initiation of appropriate initial breathing support and ensures effectiveness**<br>e.g. oxygen, bag and mask ventilation<br>- effectiveness based on ausculatation, ensuring adequate chest rise, rate	O	O	O	O	O
6. **Identifies need for and obtains appropriate airway intervention as required**<br>e.g. no intervention required, positioning, jaw thrust, oral or nasal airway, or intubation	O	O	O	O	O
7. **Ensures adequacy of airway and breathing after each intervention**<br>- auscultation<br>- adequate chest rise with bagging<br>- oxygen saturations<br>- proper endotracheal tube placement if intubated (e.g. auscultation, end tidal CO2, chest rise, tube misting)	O	O	O	O	O
8. **Asks for assessment of pulses and perfusion**<br>- asks for capillary refill time, colour, temperature	O	O	O	O	O
9. **Asks for initiation of chest compressions when appropriate and ensures adequacy of compressions**<br>- checks pulses with compressions<br>- ensures appropriate rate and depth<br>- ensures interruptions in compressions are minimized<br>- asks for change in person doing compressions every few minutes to avoid fatigue	O	O	O	O	O
10. **Ensures timely appropriate vascular access**<br>- inserts IV or confirms present IV working<br>- request IO within 90 seconds if unable to obtain IV access	O	O	O	O	O
11. **Verbally identifies cardiac rhythm on monitor and reassesses rhythm and pulse appropriately after each intervention**	O	O	O	O	O
12. **Chooses interventions according to appropriate PALS algorithm**<br>- correct medication, dose, route<br>- correct use of defibrillator (choice of none, defibrillation, or cardioversion including appropriate energy dose)	O	O	O	O	O
13. **Orders appropriate investigations**<br>- blood gas, electrolytes, glucose<br>- x-rays<br>- ECG, rhythm strip	O	O	O	O	O
14. **Asks for assessment of neurological status (e.g. pupil check) or secondary survey once stabilization of ABC's complete**	O	O	O	O	O

34660

Page 2 of 2

Fig. 1. Continued.

from KCS loaded on both Factor 1 and Factor 2. These were items dealing with use of resources, patient reassessment or advanced planning as part of their content, and therefore were felt to incorporate competencies from both "Leadership and Communication Skills" and "Knowledge and Clinical Skills" subcompetencies.

4. Discussion

This study demonstrates that a comprehensive instrument to assess pediatric resuscitation competence as a resuscitation team leader can be developed with robust psychometric
