# Supplementary material for: The impact of CPR coach presence and position on team leader and team performance during asystole simulation scenario: a randomized simulation-based trial
Source: PLoS One. 2026 Mar 12;21(3):e0344568. doi: 10.1371/journal.pone.0344568 (PMC12981441; doi:10.1371/journal.pone.0344568)
Supplement: S4 File — (PDF) [file pone.0344568.s004.pdf]

CPT (Clinical Performance Tool)

PHASE 1: ASYSTOLE.

Task	0 Point	1 Point	2 Points
Pulse check	• Not done	• >30 s<br>• Peripheral pulse<br>• After CPR started or epinephrine given	• <30 s and in sequence
CPR	• Not done	• Done without pulse check<br>• Done after epinephrine given<br>• >30 s after pulselessness recognized	• <30 s after pulselessness recognized and before epinephrine
ECG	• Not done	• Done without clinical assessment of circulation<br>• Done before CPR if pulselessness recognized<br>• Done after epinephrine<br>• >60 s	• Done after CPR started for pulselessness and before other therapy
IV/IO access	• Not done<br>• Only done once need for IV med recognized	• IV instead of IO<br>• >60 s	• IO in <60 s
Epinephrine	• Not done	• Called for without pulse check<br>• Called for without CPR<br>• Called for without via ETT<br>• >30 s after pulselessness recognized	• Called for after pulse check and CPR within 30 s of pulselessness recognition
Pulse recheck after ROSC	• Not done (includes ROSC never achieved)	• 30 s after ROSC<br>• Peripheral pulse check	• Central pulse checked within 30 s of ROSC
Defibrillation	• Called for	• Never called for	

CPR, cardiopulmonary resuscitation; ECG, electrocardiography; ETT, endotracheal tube; med, medicine; ROSC, return of spontaneous circulation.
Scoring instrument for asystole scenario (example, with permission from Donoghue et al[3]).
Scoring instrument for other scenarios (with permission from Donoghue et al[3]).
