# Supplementary material for: The impact of CPR coach presence and position on team leader and team performance during asystole simulation scenario: a randomized simulation-based trial
Source: PLoS One. 2026 Mar 12;21(3):e0344568. doi: 10.1371/journal.pone.0344568 (PMC12981441; doi:10.1371/journal.pone.0344568)
Supplement: S5 File — (PDF) [file pone.0344568.s005.pdf]

Regione del Veneto – Azienda Ospedale Università di Padova

Ethics Committee for Clinical Trials of the Province of Padua

Minutes of December 15, 2022

Meeting of the Ethics Committee for Clinical Trials of the Province of Padua, appointed by Resolution of the General Director No. 1269 dated 23/10/2019, and subsequent Resolutions of the General Director, executive pursuant to law, operating under Ministerial Decree 08/02/2013 and in accordance with ICH-GCP principles, held via teleconference on December 15, 2022, at 14:30.

Member	Role	Presence
Dr. Francesca Bano – Pharmacist AULSS 6 Euganea (I)	Absent	
Dr. Gianni Binotto – Clinician “Additional expert” AOP (I)	Absent	
Dr. Giulia Cuman – Expert in Bioethics (E)	Present	
Dr. Ilaria de Barbieri – Nurse AOP (I)	Present	
Lawyer Erica De Candido Romole – Jurist (E)	Present	
Dr. Federica De Lotto – Forensic Physician (E)	Present	
Dr. Guido Di Gregorio – Clinician AULSS 6 Euganea (I)	Absent	
Prof. Marco Di Pascoli – Clinician AOP (I)	Present	
Dr. Vera Frison – Clinician AULSS 6 Euganea (I)	Present	
Dr. Nicola Gennaro – Biostatistician (E)	Present	
Dr. Paolo Girardi – Biostatistician (E)	Present	
Dr. Cecilia Giron – Clinical Pharmacologist (E)	Present	
Dr. Annamaria Grion – Pharmacist “Additional expert” (E)	Present	
Prof. Gino Marioni – Clinician “Additional expert” AOP (I)	Absent	
Dr. Diego Martines – Clinician “Additional expert” (E)	Present	
Dr. Francesca Mungo – General Practitioner (E)	Present	
Dr. Linda Nicolardi – Clinician AULSS 6 Euganea (I)	Present	
Dr. Anna Redomi – Expert in Medical Devices AOP (I)	Present	

Member	Role	Presence
Dr. Lorenza Sanavio – Representative of the Voluntary Sector for patient assistance or protection associations, designated by the Volunteer Service Center (E)	Absent	
Dr. Marco Simonetto – Clinician AULSS 6 Euganea (I)	Present	
Prof. Claudio Terranova – Forensic Physician AOP (I)	Present	
Dr. Alvise Tosoni – Pediatrician AOP (I)	Present	
Dr. Francesca Venturini – Pharmacist AOP (I)	Present	

Member	Role	Presence
Dr. Alfio Capizzi – Delegate of the Health Director, Azienda Ospedaliera di Padova (I)	Absent	
Dr. Michela Galdarossa – Delegate of the Health Director, AULSS 6 Euganea (I)	Absent	
Expert	Role	Presence
Dr. Massimo Napodano – Clinical expert in relation to studies of new technical, diagnostic, and therapeutic, invasive and semi-invasive procedures AOP (I)	Present	

Legend: (I) Internal Member (E) External Member

Dr. Federica De Lotto chairs the session.

The attending members agree not to express opinions on studies in which they may have a direct or indirect conflict of interest and commit, should such a conflict arise during review, to abstain from voting and to leave the meeting.

For pediatric studies, the opinion was expressed after consultation with Dr. Alvise Tosoni.

Field	Details
**CESC Code**	/
**URC Code**	/
**Study Code / EudraCT Number**	/
**Investigator**	Prof. Roberto Vettor

Field	Details
**Structure**	Advanced Simulation Unit SimulARTI, University of Padua - DIMED
**Title**	Non-profit, monocentric, randomized clinical investigation of medical/surgical procedures on the introduction of a CPR coach figure in simulation and its influence on team leader and team performance – *CPR Coaching on Leader's Performance Study*
**Active Ingredient / Device**	/
**Protocol Entry Number / Date**	81202 – 12/12/2022
**Date of Acknowledgment**	12/12/2022
**Documents Reviewed**	- Letter dated 07/12/2022 requesting acknowledgment by Prof. Roberto Vettor - Study Protocol - Study Synopsis, version 1.0 dated 29/10/2022 - Data Collection Sheet, version 1.0 dated 29/10/2022 - Information Sheet and Informed Consent Form - Information Notice and Consent Form pursuant to Article 13 of Regulation (EU) 2016/679, version 1.0 dated 29/10/2022
**Opinion**	The Committee acknowledges receipt.

The meeting adjourned at 17:47.

The Vice President of the Ethics Committee for Clinical Trials

(signed) Dr. Federica De Lotto

Certified copy of the original

Head of the Scientific Secretariat

Dr. Camillo Barbisan

(Digitally signed document)
