# Supplementary material for: The impact of CPR coach presence and position on team leader and team performance during asystole simulation scenario: a randomized simulation-based trial
Source: PLoS One. 2026 Mar 12;21(3):e0344568. doi: 10.1371/journal.pone.0344568 (PMC12981441; doi:10.1371/journal.pone.0344568)
Supplement: S6 File — (PDF) [file pone.0344568.s006.pdf]

Protocollo di studio CPR coach EESOA – Università di Padova

Introduzione: Recentemente nell'ambito delle linee guida 2020 AHA sulla rianimazione cardio polmonare è stata introdotta la figura professionale del CPR Coach la cui responsabilità principale è quella di fornire in tempo reale un feedback delle prestazioni del team di rianimazione durante l'arresto cardiaco, permettendo così al team leader di concentrarsi sul supporto vitale avanzato e sulla gestione delle cause reversibili. La figura del Coach deve coordinare l'inizio della RCP, comunicare i dati dei dispositivi di feedback per aiutare chi esegue le compressioni nel migliorare la performance, comunicare i target medi delle compressioni e ventilazioni, coordinare le operazioni per raggiungere tali valori e comunicare con il team per ridurre le pause delle compressioni.

In studi precedenti (Hunt et al,2018, Cheng et al,2018) si è visto come l'introduzione del ruolo del CPR Coach nei team di rianimazione ospedaliera abbia portato ad un aumento dell'aderenza della RCP alle linee guida AHA da parte del team.

Ad oggi rimane ancora poco studiata l'interazione tra la figura del CPR Coach e del Team Leader durante l'esecuzione di una RCP.

In questo studio ci proponiamo di valutare se l'interazione tra il CPR Coach e il Team leader porti dei benefici reali all'impegno cognitivo di quest'ultimo e quindi se venga inficiata o meno la sua leadership e la performance globale del team.

Descrizione dello studio: In questo studio prospettico randomizzato viene suddivisa la popolazione di studio in tre gruppi A, B e C. I gruppi A e B saranno composti da squadre di 6 partecipanti ciascuna costituite da: due componenti della CPR, un team leader, un CPR coach, un attore infermiere e un attore al defibrillatore.

Le squadre del gruppo controllo C, in cui non è presente la figura del coach, saranno costituite da 6 partecipanti: tre componenti della CPR, un team leader, un attore infermiere e un attore al defibrillatore. I partecipanti di ciascuna squadra saranno scelti in maniera randomica.

Ciascuna squadra eseguirà una simulazione di rianimazione cardio polmonare (CPR) di 10 minuti su un manichino standardizzato Trauma Hal mannequin (Gaumard Scientific) con un Combat Application Tourniquet (CAT Resources, Rock Hill, SC)/SUSIE simulator(Gaumard Scientific, Miami, FL).

Tutti i partecipanti a cui sarà assegnato il ruolo di CPR coach riceveranno un addestramento standardizzato basato su linee guida AHA 2020.

Il gruppo di studio A eseguirà una simulazione con la figura del CPR coach in squadra posizionato accanto al defibrillatore; il gruppo di studio B eseguirà una simulazione con la figura del CPR coach in squadra libero di muoversi nello scenario; il gruppo di controllo C eseguirà una simulazione senza la figura del CPR coach in squadra.

Durante le simulazioni, un facilitatore esperto e un tecnico di simulazione saranno presenti per condurre e sorvegliare l'andamento delle attività stesse.
Tutte le attività di tutte le squadre verranno registrate con telecamere e microfoni.
Alla fine di ciascuna simulazione ci sarà un debriefing condotto da un facilitatore esterno per rendere l'esperienza simulativa completa ma non sarà preso in considerazione nello studio.
Tutti i video registrati saranno visionati da tre osservatori esperti indipendenti che assegneranno un punteggio alla performance del leader e alla performance della squadra. Tutti gli sperimentatori saranno preliminarmente valutati per grado di concordanza tra osservatori indipendenti (inter-observer reliability).

Ipotesi nulla H0:

1. L'introduzione della figura di un coach nella CPR in simulazione migliora la performance del team ma influisce sulla performance del team leader.
2. La posizione del CPR coach influisce sull'outcome della squadra e del team leader.

Endpoint primario: Valutazione performance del leader (leadership)

Endpoint secondario: Valutazione performance del team

Rilevazione degli Endpoint:

Endpoint primario: Performance del team leader (Leadership) Resuscitation Team Leader Evaluation Scale. **(FIG 1)**
Grant EC, Grant VJ, Bhanji F, Duff JP, Cheng A, Lockyer JM. The development and assessment of an evaluation tool for pediatric resident competence in leading simulated pediatric resuscitations. Resuscitation. 2012 Jul;83(7)

Endpoint secondario: Valutazione Performance del team:

- CPT (Clinical Performance Tool) **(FIG 2)** Levy A, Donoghue A, Bailey B, Thompson N, Jamoulle O, Gagnon R, Gravel J. External validation of scoring instruments for evaluating pediatric resuscitation. Simul Healthc. 2014 Dec;9(6):360-9.

- qualità esecuzione RCP (**software interno manichino)**
    - Chest compression fraction >60% (minimize interruptions)
    - Compression rate of 100-120/min.
    - Compression depth between 50 to 60 mm Full chest recoil > 75%
    - Avoid excessive ventilation (< 12 breaths per minute)

    Punteggio 1 se raggiunto l'obiettivo, 0 se non raggiunto REF ILA

Partecipanti allo studio: Medici specializzandi di Medicina D'Emergenza, Medicina interna e di Anestesia e Rianimazione (2 anno con esperienza in rianimazione? BLSD

Dimensione del campione: In base alla power analysis determinata sull'endpoint primario dello studio è necessario un campione di 121 partecipanti per assicurare un livello di significatività del 95% e un potere del 80%. I partecipanti saranno randomizzati in 3 Gruppi (A,B,C) di 11 squadre ciascuno. La composizione delle squadre è stata descritta nella sezione "Descrizione dello studio".

Durata: 6-12 mesi

Registrazione: Lo studio è stato registrato su ClinicalTrials.gov con il numero identificativo: NCT05309434

Ref:

Hunt EA, Jeffers J, McNamara L, Newton H, Ford K, Bernier M, Tucker EW, Jones K, O'Brien C, Dodge P, Vanderwagen S, Salamone C, Pegram T, Rosen M, Griffis HM, Duval-Arnould J. Improved Cardiopulmonary Resuscitation Performance With CODE ACES2: A Resuscitation Quality Bundle. J Am Heart Assoc. 2018 Dec 18;7(24)

Cheng A, Duff JP, Kessler D, Tofil NM, Davidson J, Lin Y, Chatfield J, Brown LL, Hunt EA; International Network for Simulation-based Pediatric Innovation Research and Education (INSPIRE) CPR. Optimizing CPR performance with CPR coaching for pediatric cardiac arrest: A randomized simulation-based clinical trial. Resuscitation. 2018 Nov;132:33-40.

Donoghue A, Nishisaki A, Sutton R, Hales R, Boulet J. Reliability and validity of a scoring instrument for clinical performance during

pediatric advanced life support simulation scenarios. Resuscitation 2010;

Schema Protocollo:

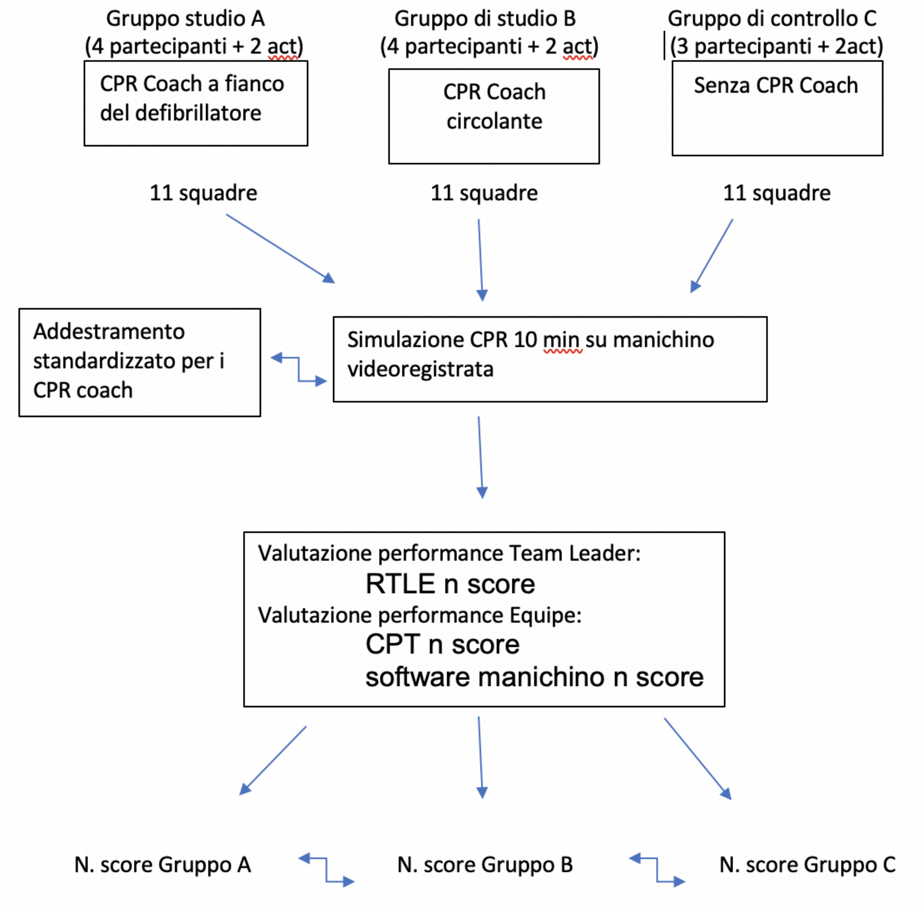

FIG. 1 *Performance del team leader (Leadership) Resuscitation Team Leader Evaluation Scale*

FACULTY OF MEDICINE | UNIVERSITY OF CALGARY

Resuscitation Team Leader Evaluation

STUDY NUMBER	ASSESSOR

Example ○ ● Please use an ink pen

Please rate the team leader on the performance statements according to the following scale.
Please use 'N/A' if the statement is not relevant to the scenario.

Part I: Leadership and Communication Skills

The team leader...	Not Performed/ Not Observed 0	Performed, but ineffectively, incompletely or inconsistently 1	Performed adequately most of the time 2	Performed well consistently 3	Item not relevant to scenario N/A
1. **Clearly identifies he/she will lead the resuscitation**	○	○	○	○	○
2. **Delegates roles and responsibilities to team members**	○	○	○	○	○
3. **Maintains control of leading the resuscitation**<br>- manages distractions<br>- avoids allowing others to give orders<br>- controls noise and crowd	○	○	○	○	○
4. **Uses effective closed loop communication**<br>- questions and orders are given clearly and assertively<br>- person spoken to is identified by name or other clear method<br>- ensures team member heard and understood (e.g. through member's response to question or by asking for verbal confirmation once order completed)	○	○	○	○	○
5. **Manages team resources and distributes workload appropriately**<br>- avoids overloading or underloading team members<br>- avoids giving multiple orders at once<br>- prioritizes multiple orders when several are needed	○	○	○	○	○
6. **Verbalizes thoughts and summarizes progress periodically for benefit of team (shares situational awareness and mental models)**<br>- describes events so far<br>- states suspected diagnosis as well as other possibilities	○	○	○	○	○
7. **Asks for and acknowledges input from team members**<br>- asks for other ideas<br>- asks for confirmation of suspected diagnosis<br>- incorporates ideas from team when appropriate	○	○	○	○	○
8. **Reassesses and reevaluates situation frequently**<br>- verbally identifies changes in patient status in a timely fashion<br>- acknowledges changes in status identified by team members	○	○	○	○	○
9. **Avoids fixation errors (getting "stuck" on a particular issue)**<br>- acknowledges information that is inconsistent with interpretation<br>- uses new information or changes in status as an opportunity to reconsider other diagnoses<br>- reassesses situation when interventions not producing desired effect	○	○	○	○	○
10. **Refrains if possible from active participation (hands-off)**	○	○	○	○	○
11. **Shows anticipation of future events by asking for preparation of equipment or medication not yet needed**<br>- asks for x-ray to be called ahead of when ready<br>- asks for infusions to be mixed up before needed	○	○	○	○	○
12. **Asks for appropriate help early and shows awareness of own limitations.**<br>- asks for additional personnel for extra hands<br>- asks for consultants to be called for advice	○	○	○	○	○

34660

Page 1 of 2

Part II: Knowledge and Clinical Skills

The team leader...	Not Performed/ Not Observed 0	Performed, but ineffectively or incompletely 1	Performed effectively but delayed or out of sequence 2	Performed well in a timely manner 3	Item not relevant to scenario N/A
1. **Obtains preliminary history quickly or designates other to do so**	○	○	○	○	○
2. **Obtains full cardiorespiratory monitoring and full set of vitals promptly**<br>- HR, RR, BP, Sat, Temp	○	○	○	○	○
3. **Obtains assessment of airway patency and protection**<br>e.g. - establishes if patient verbalizing<br>- checks for open airway<br>- identifies protection of airway based on neurological status	○	○	○	○	○
4. **Obtains assessment of breathing adequacy**<br>- auscultation<br>- assessment of work of breathing<br>- adequacy of ventilation (rate, depth)	○	○	○	○	○
5. **Asks for initiation of appropriate initial breathing support and ensures effectiveness**<br>e.g. oxygen, bag and mask ventilation<br>- effectiveness based on ausculatation, ensuring adequate chest rise, rate	○	○	○	○	○
6. **Identifies need for and obtains appropriate airway intervention as required**<br>e.g. no intervention required, positioning, jaw thrust, oral or nasal airway, or intubation	○	○	○	○	○
7. **Ensures adequacy of airway and breathing after each intervention**<br>- auscultation<br>- adequate chest rise with bagging<br>- oxygen saturations<br>- proper endotracheal tube placement if intubated (e.g. auscultation, end tidal CO2, chest rise, tube misting)	○	○	○	○	○
8. **Asks for assessment of pulses and perfusion**<br>- asks for capillary refill time, colour, temperature	○	○	○	○	○
9. **Asks for initiation of chest compressions when appropriate and ensures adequacy of compressions**<br>- checks pulses with compressions<br>- ensures appropriate rate and depth<br>- ensures interruptions in compressions are minimized<br>- asks for change in person doing compressions every few minutes to avoid fatigue	○	○	○	○	○
10. **Ensures timely appropriate vascular access**<br>- inserts IV or confirms present IV working<br>- request IO within 90 seconds if unable to obtain IV access	○	○	○	○	○
11. **Verbally identifies cardiac rhythm on monitor and reassesses rhythm and pulse appropriately after each intervention**	○	○	○	○	○
12. **Chooses interventions according to appropriate PALS algorithm**<br>- correct medication, dose, route<br>- correct use of defibrillator (choice of none, defibrillation, or cardioversion including appropriate energy dose)	○	○	○	○	○
13. **Orders appropriate investigations**<br>- blood gas, electrolytes, glucose<br>- x-rays<br>- ECG, rhythm strip	○	○	○	○	○
14. **Asks for assessment of neurological status (e.g. pupil check) or secondary survey once stabilization of ABC's complete**	○	○	○	○	○

34660

Page 2 of 2

FIG. 2 *CPT (Clinical Performance Tool)*

PHASE 1: ASYSTOLE.

Task	0 Point	1 Point	2 Points
Pulse check	• Not done	• >30 s<br>• Peripheral pulse<br>• After CPR started or epinephrine given	• <30 s and in sequence
CPR	• Not done	• Done without pulse check<br>• Done after epinephrine given<br>• >30 s after pulselessness recognized	• <30 s after pulselessness recognized and before epinephrine
ECG	• Not done	• Done without clinical assessment of circulation<br>• Done before CPR if pulselessness recognized<br>• Done after epinephrine<br>• >60 s	• Done after CPR started for pulselessness and before other therapy
IV/IO access	• Not done<br>• Only done once need for IV med recognized	• IV instead of IO<br>• >60 s	• IO in <60 s
Epinephrine	• Not done	• Called for without pulse check<br>• Called for without CPR<br>• Called for without via ETT<br>• >30 s after pulselessness recognized	• Called for after pulse check and CPR within 30 s of pulselessness recognition
Pulse recheck after ROSC	• Not done (includes ROSC never achieved)	• 30 s after ROSC<br>• Peripheral pulse check	• Central pulse checked within 30 s of ROSC
Defibrillation	• Called for	• Never called for	

CPR, cardiopulmonary resuscitation; ECG, electrocardiography; ETT, endotracheal tube; med, medicine; ROSC, return of spontaneous circulation.
Scoring instrument for asystole scenario (example, with permission from Donoghue et al[3]).
Scoring instrument for other scenarios (with permission from Donoghue et al[3]).

versione 1.0 del 29/10/2022
