# Supplementary material for: The impact of CPR coach presence and position on team leader and team performance during asystole simulation scenario: a randomized simulation-based trial
Source: PLoS One. 2026 Mar 12;21(3):e0344568. doi: 10.1371/journal.pone.0344568 (PMC12981441; doi:10.1371/journal.pone.0344568)
Supplement: S7 File — (PDF) [file pone.0344568.s007.pdf]

Study Protocol – CPR Coach EESOA – University of Padua

Introduction

The 2020 AHA Guidelines for Cardiopulmonary Resuscitation recently introduced the professional role of the **CPR Coach**, whose main responsibility is to provide real-time feedback on resuscitation team performance during cardiac arrest, allowing the team leader to focus on advanced life support and the management of reversible causes.

The CPR Coach coordinates the initiation of CPR, communicates device feedback to support compressors in improving performance, communicates target metrics for compressions and ventilations, coordinates actions to achieve these values, and interacts with the team to reduce interruptions in chest compressions.

Previous studies (Hunt et al., 2018; Cheng et al., 2018) demonstrated that introducing the CPR Coach role in in-hospital resuscitation teams increases adherence to AHA guidelines.

To date, **the interaction between the CPR Coach and the Team Leader during CPR remains understudied**.

This study aims to assess whether interaction between the CPR Coach and the Team Leader provides real cognitive benefit to the Team Leader and whether their leadership and overall team performance are affected.

Study Description

This prospective randomized study divides participants into three groups: **A, B, and C**.

Groups **A** and **B** will consist of 6 participants each:

- Two compressors
- One team leader
- One CPR coach
- One nurse actor
- One defibrillator operator actor

Control group **C** will include 6 participants **without a CPR coach**:

- Three compressors
- One team leader
- One nurse actor
- One defibrillator operator actor

Participants will be randomly assigned to teams.

Each team will perform a **10-minute CPR simulation** on a standardized Trauma HAL mannequin (Gaumard Scientific) with a Combat Application Tourniquet (CAT Resources, Rock Hill, SC) / SUSIE simulator (Gaumard Scientific, Miami, FL).

All participants assigned as CPR Coaches will receive standardized training based on the **2020 AHA Guidelines**.

- **Group A**: CPR Coach positioned near the defibrillator
- **Group B**: CPR Coach allowed to move freely within the scenario
- **Group C**: Simulation **without CPR Coach**

A simulation facilitator and a simulation technician will supervise all scenarios.

All simulations will be **audio-video recorded**.

After each session, a **debriefing** will be conducted for educational purposes, but **will not be analyzed** in the study.

Recordings will be reviewed by **three independent expert observers**, who will score team leader performance and team performance.

All raters will be evaluated for **inter-observer reliability** before data analysis.

Null Hypotheses (H0)

- The introduction of a CPR Coach in simulation improves overall team performance but affects Team Leader performance.
- The position of the CPR Coach affects team and Team Leader outcomes.

Primary Endpoint

- **Team Leader performance (leadership)**

Secondary Endpoint

- **Team performance**

Endpoint Measurement

Primary Endpoint

Team Leader performance measured using:

Resuscitation Team Leader Evaluation Scale

Grant EC, Grant VJ, Bhanji F, Duff JP, Cheng A, Lockyer JM. *Resuscitation* 2012;83(7).

Secondary Endpoint

Team performance measured using:

- **CPT (Clinical Performance Tool)**

  Levy A et al. *Simul Healthc.* 2014;9(6):360-9

- **Quality of CPR execution** (manikin software)
  - Chest compression fraction >60%
  - Compression rate 100–120/min
  - Compression depth 50–60 mm
  - Full chest recoil >75%
  - Avoid excessive ventilation (<12 breaths/min)

Scoring: 1 if target achieved, 0 if not achieved

Participants

Residents in:

- Emergency Medicine
- Internal Medicine
- Anesthesiology and Intensive Care

(2nd-year trainees with CPR/ALS course experience)

Sample Size

Based on power analysis for the primary endpoint, **121 participants** are required ($\alpha=0.05$, power=80%).

Participants will be randomized into **three groups (A, B, C)** of **11 teams each**.

Team composition described above.

Duration

6–12 months

Registration

The study is registered on **ClinicalTrials.gov**:

NCT05309434
